# Detection of food-borne bacteria in ready to eat betel leaf sold at local markets in Mymensingh

**DOI:** 10.14202/vetworld.2017.1040-1045

**Published:** 2017-09-11

**Authors:** Md. Mazedul Haque, Md. Atiqur Rahman Sarker, Rafia Afroze Rifa, Md. Ariful Islam, Mst. Minara Khatun

**Affiliations:** Department of Microbiology and Hygiene, Faculty of Veterinary Science, Bangladesh Agricultural University, Mymensingh 2202, Bangladesh

**Keywords:** betel leaf, food-borne bacteria, Mymensingh city, public health importance

## Abstract

**Aim:**

The present study was undertaken to determine bacterial load as well as characterize bacterial flora of ready to eat (RTE) betel leaf sold at local markets in Mymensingh city.

**Materials and Methods:**

A total of 25 RTE betel leaf samples were collected from five local markets such as Kamal-Ranjit (KR) market, Shesh more, Kewatkhali, Jobber more, and Ganginar par.

**Results:**

Total viable count of bacteria in betel leaf (log_10_ mean colony forming unit±standard deviation/ml) was 7.58±0.04 for KR market, 7.72±0.06 for Shesh more, 7.62±0.04 for Kewatkhali, 7.40±0.03 for Jobber more, and 7.60±0.06 for Ganginar par. A total of 98 bacterial isolates belong to five genera (*Escherichia coli*, *Salmonella* spp., *Vibrio* spp., *Bacillus* spp., and *Staphylococcus* spp.) were identified. The prevalence of *E. coli* was 17.34%, *Salmonella* spp. was 25.51%, *Vibrio* spp. was 19.39%, *Bacillus* spp. was 18.37%, and *Staphylococcus* spp. was 19.39%. Antibiotic sensitivity test showed that all isolates were sensitive to two antibiotics such as ciprofloxacin and gentamicin. Four isolates (*E. coli*, *Salmonella* spp., *Vibrio* spp., and *Staphylococcus* spp.) were resistant to two antibiotics (ampicillin and cephalexin). Antibiogram profile of bacterial isolates of betel leaf suggests that they were multidrug resistance.

**Conclusion:**

Data of this study indicate that betel leaf sold at local market harbors multidrug resistance food-borne bacteria which might cause public health hazards if these antibiotic resistant transfer to human through food chain.

## Introduction

Betel leaf commonly known as paan belongs to the family *Piperaceae* [1], and the scientific name is *Piper betle* [2]. Paan is extensively grown in Bangladesh, India, Sri Lanka, Malaysia, Indonesia, Philippines, and East Africa [3]. In Bangladesh, huge numbers of peoples consume paan daily which is more common in people aged 35-60 years. The significance of the leaves has been explained in relation to every sphere of human life including social, cultural, religious, medicinal, and even daily life such as marriage, puja, and Sraddha ceremony.

Bangladesh earns US$ 8 million per year by exporting betel leaf to European country which contributes a great to our national economy [4]. However, the European Union (EU) could detect the contaminated *Salmonella* in Bangladeshi betel leaves which was harmful for health and caused diarrhea and vomiting and other related serious illness to the people who consumed it, and ultimately, the EU stopped the importation of betel leaf from Bangladesh [5]. Export of paan leaves from Bangladesh is under a great threat. Our previous studies showed the efficacy of vinegar, sorbitol, and sodium benzoate in mitigation of *Salmonella* contamination in betel leaf [6]. Research work on the isolation, identification, and characterization of bacteria from the betel leaf has not been conducted in Bangladesh.

The aim of this study was to determine bacterial load as well as to isolate and identify bacteria from betel leaves collected from different local markets of Mymensingh.

## Materials and Methods

### Ethical approval

The experiment was approved by Institutional Ethical Committee, Faculty of Veterinary Science, Bangladesh Agricultural University, Mymensingh 2202, Bangladesh.

### Collection and transportation of betel leaf (paan) samples

A total of 25 betel leaf samples were collected from five different local markets such as Kamal-Ranjit (KR) market, Shesh more, Kewatkhali, Jobber more, and Ganginar par in Mymensingh (Table-1). Individual sample was placed in the sterile polythene bag. The samples were transported carefully to the bacteriology laboratory using an icebox for bacteriological analysis.

**Table-1:** Summary of betel leaf samples collected from local markets.

Name of markets	Number of betel leaf collected from each market	Total
KR	5	25
Shesh more	5	
Kewatkhali	5	
Jobber more	5	
Ganginar par	5	

KR=Kamal-Ranjit

### Washing of betel leaf samples

The betel leaf samples in polythene bag were washed with sterile phosphate-buffered saline (PBS). One betel leaf was washed with 20 ml of sterile PBS. A 10-fold serial dilution of the washed samples was prepared in nutrient broth. Five betel leaf samples from each market were washed and analyzed for isolation of bacteria.

### Determination of total viable count (TVC)

A total of 0.5 ml 10-fold diluted sample (10^−1^ to 10^−7^) was transferred and spreaded onto plate count agar and incubated at 37°C for 24-48 h. The number of colonies (30-300) in a particular dilution was multiplied by the dilution factor to calculate TVC which was expressed as mean log_10_±standard deviation colony-forming unit (CFU)/ml.

### Isolation and identification of bacteria

Isolation and identification of bacteria were performed according to the method described by Carter [7]. Washing of betel leaf was enriched in nutrient broth at 37°C for overnight. The overnight cultures were streaked on eosin methylene blue (EMB) agar for *Escherichia coli*, *Salmonella*-*Shigella* agar or xylose-lysine deoxycholate (XLD) agar for *Salmonella* spp., thiosulfate-citrate-bile salts-sucrose (TCBS) agar for *Vibrio* spp., blood agar for *Bacillus* spp., and mannitol salt (MS) agar for *Staphylococcus* spp. and incubated at 37°C for 24 h. Single colony was further subcultured until pure culture was obtained. Identification of bacteria was performed on the basis of colony morphology; Gram’s staining reaction, motility test, and biochemical tests.

### Molecular detection of E. coli by polymerase chain reaction (PCR)

#### DNA extraction

A pure bacterial colony of *E. coli* was mixed with 100 µl of distilled water which was boiled for 10 min then immediately kept on ice for cold shock. Finally, centrifugation was done at 10,000 rpm for 10 min. The supernatant was collected and used as DNA template for PCR.

### Primers used for PCR

A genus-specific PCR was performed to amplify 16S rRNA of *E. coli* using previously published primers [8]. The list of primers is shown in Table-2.

**Table-2:** PCR primers with sequence.

Primer	Sequence	Size (bp)
*E. coli* 16S (F)	5’-AATTGAAGAGTTTGATCATG-3’	704
*E. coli* 16S (R)	5’-CTCTACGCATTTCACCGCTAC-3’	

F=Forward, R=Reverse, bp=Base pair, PCR=Polymerase chain reaction, *E. coli*=*Escherichia coli*

### Antibiotic sensitivity test

Five isolates randomly selected from five genera were tested for antimicrobial drug susceptibility against five commonly used antibiotics such as ampicillin, chloramphenicol, ciprofloxacin, gentamicin, and cephalexin by disc diffusion or Kirby-Bauer method [9]. Antimicrobial testing results were recorded as susceptible, intermediate, and resistant according to zone diameter interpretative standards provided by the Clinical and Laboratory Standards Institute [10].

### Statistical analysis

The results of TVC of bacteria of betel leaf sold at local markets were analyzed for statistical significance using Duncan’s multiple range test (SPSS, 11.5). A p<0.05 was considered to be statistically significant.

## Results

### TVC of betel leaf

**Figure-1:**
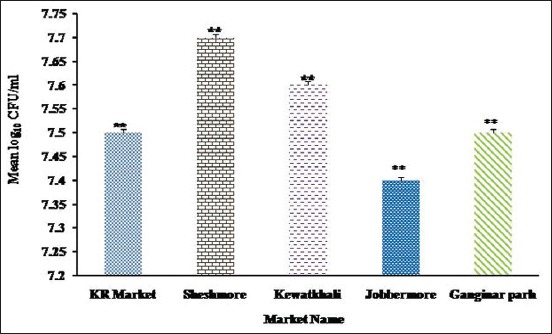
Total viable counts of bacteria recovered from betel leaf sold at Kamal-Ranjit markets, Shesh more, Kewatkhali, Jobber more, and Ganginar par markets. The result is expressed as mean log_10_ colony-forming unit of 5 samples ± standard deviation/ml. The bacterial load recorded in betel leaf sold in all local markets was found to be statistically significant (**p<0.01).

**Figure-2:**
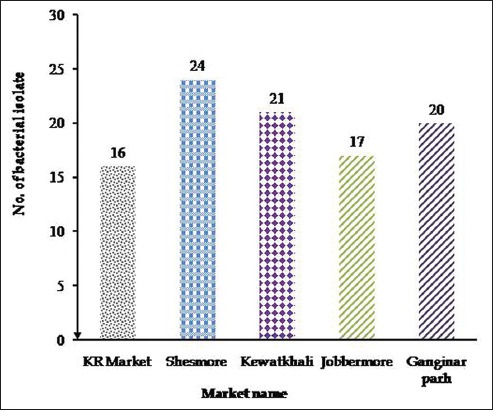
Number of bacterial isolates recovered from betel leaf sold at Kamal-Ranjit (KR) market, Shesh more, Kewatkhali, Jobber more, and Ganginar par. The highest number of bacteria was recovered from Shesh more (24 isolates from 5 samples), followed by Kewatkhali (21 isolates from 5 samples), Jobber more (17 isolates from 5 samples), Ganginar par (20 isolates from 5 samples), and KR market (16 isolates from 5 samples).

The TVC of betel leaf samples collected from different markets is presented in Figure-1. A number of bacterial isolates recovered from betel leaf sold at different markets are shown in Figure-2.

### Isolation of bacteria from betel leaf

A total of 98 bacterial isolates belonged to five genera of bacteria such as *E. coli*, *Salmonella* spp., *Vibrio* spp., *Bacillus* spp., and *Staphylococcus* spp. were isolated from 25 betel leaf samples (Table-3).

**Table-3:** Summary of isolation of bacteria from betel leaf sold at local markets in Mymensingh.

Name of local markets	Bacterial genera				

	*E. coli* (n)	*Salmonella* spp. (n)	*Vibrio* spp. (n)	*Bacillus* spp. (n)	*Staphylococcus* spp. (n)
KR market	4	5	4	3	ND
Shesh more	5	5	4	5	5
Kewatkhali	4	5	3	4	5
Jobber more	ND	5	4	3	5
Ganginar par	4	5	4	3	4
Total	17	25	19	18	19

KR=Kamal-Ranjit, ND=Not detected, *E. coli*=*Escherichia coli*

### Results of biochemical test

Results of sugar fermentation tests using five basic sugars such as dextrose, maltose, lactose, sucrose, and mannitol and other biochemical tests such as methyl red (MR) and Voges–Proskauer (VP) are listed in Table-4.

**Table-4:** Biochemical characteristics of *E. coli*, *Salmonella* spp., *Vibrio* spp., *Bacillus* spp., and *Staphylococcus* spp.

Sugar fermentation reaction profiles					MR test	VP test	Indole production test	Catalase test	Interpretation

DX	ML	L	S	MN					
AG	AG	AG	AG	AG	+	-	+	-	*E.coli*
A	A	-	-	A	+	-	-	-	*Salmonella* spp.
A	A	-	A	A	+	-	+	+	*Vibrio* spp.
AG	A	A	AG	AG	+	-	+	-	*Bacillus* spp.
A	A	A	A	A	+	+	-	+	*Staphylococcus* spp.

DX=Dextrose, ML=Maltose, L=Lactose, S=Sucrose, MN=Mannitol, A=Acid, AG=Acid and gas, +=Positive, -=Negative, MR=Methyl red, VP=Voges–Proskauer, *E.coli=Escherichia coli*

In sugar fermentation test, *E. coli* fermented all five basic sugars such as dextrose, maltose, lactose, sucrose, and mannitol and produced acid and gas. *Salmonella* spp. fermented dextrose, maltose, and mannitol and produced only acid, whereas lactose and sucrose were negative. *Vibrio* spp. fermented dextrose, maltose, sucrose, and mannitol and produced acid, whereas lactose was negative. *Bacillus* spp. fermented dextrose, sucrose, and mannitol and produced acid and gas. *Bacillus* spp. also fermented maltose and lactose and produced only acid. *Staphylococcus* spp. fermented all sugars and produced acid.

All isolates of *E. coli* were MR and Indole positive but VP and catalase negative; *Salmonella* was MR positive but VP, indole, and catalase negative; *Vibrio* was MR, indole, and catalase positive but VP negative; and *Bacillus* was MR and indole positive but VP and catalase negative.

### Prevalence of E. coli, Salmonella spp., Vibrio spp., Bacillus spp., and Staphylococcus spp. recovered from betel leaf

**Figure-3:**
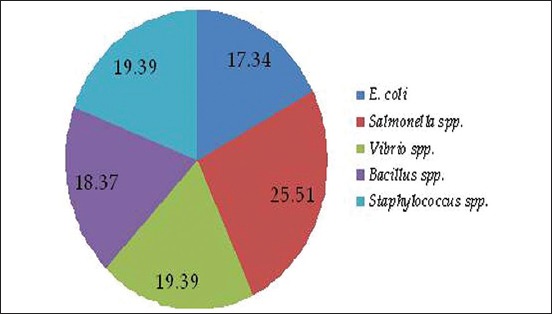
The prevalence of *Escherichia coli, Salmonella* spp., *Vibrio* spp., *Bacillus* spp., and *Staphylococcus* spp.

The prevalence of *E. coli* was 17.34% (17 of 98), *Salmonella* spp. was 25.51% (25 of 98), *Vibrio* spp. was 19.39% (19 of 98), *Bacillus* spp. was 18.37% (18 of 98), and *Staphylococcus* spp. was 19.39 (19 of 98). The overall prevalence of *E. coli, Salmonella* spp.*, Vibrio* spp.*, Bacillus* spp., and *Staphylococcus* spp. in betel leaf samples is presented in Figure-3.

### Molecular detection of E. coli

**Figure-4:**
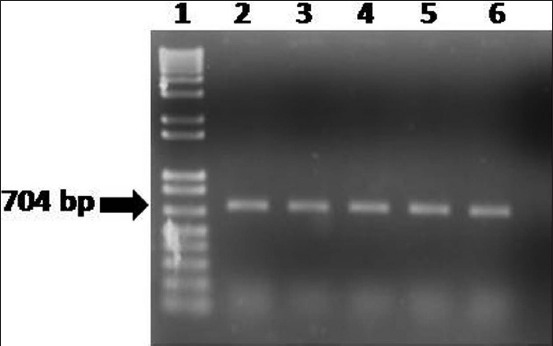
Polymerase chain reaction assay to amplify 16S rRNA of *Escherichia coli* isolates recovered from betel leaf sold at five local markets in Mymensingh. Lanes: 1 - 100-12 kb size DNA marker (Trackit, Invitrogen, USA); 2 - DNA of *E. coli* isolate of betel leaf sold at Kamal-Ranjit market; 3 - DNA of *E. coli* isolates of betel leaf sold at Shesh more market; 4 - DNA of *E. coli* isolates of betel leaf sold at Kewatkhali market; 5 - DNA of *E. coli* isolates of betel leaf sold at Jobber more; 6 - DNA of *E. coli* isolates of betel leaf sold at Ganginar par market; and 7 - Negative control without DNA.

DNA extracted from five *E. coli* isolates was used in the PCR assay. PCR primers targeting 16S rRNA of *E. coli* amplified 704 bp fragments of DNA confirming the identity of *E. coli* (Figure-4).

### Results of antibiotics sensitivity tests

**Figure-5:**
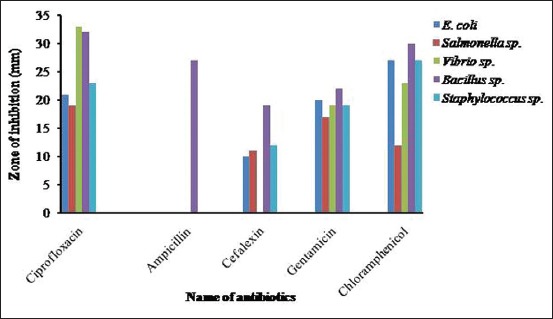
Zone of inhibition of ciprofloxacin, ampicillin, cephalexin, gentamicin, and chloramphenicol against *Escherichia coli*, *Salmonella* sp., *Vibrio* sp., *Bacillus* sp., and *Staphylococcus* sp.

One isolate randomly selected from each genus *(E. coli, Salmonella* sp., *Vibrio* sp., *Bacillus* sp., and *Staphylococcus* sp.) was subjected to antibiotic sensitivity test against five commonly used antibiotics such as ciprofloxacin, ampicillin, cephalexin, gentamicin, and chloramphenicol. The zone of inhibition of five antibiotics against tested bacteria is shown in Figure-5.

**Figure-6:**
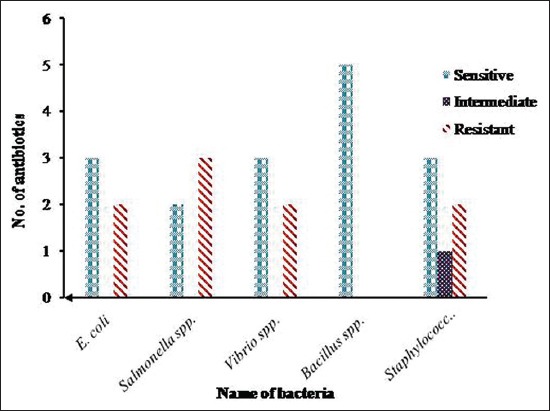
Summary of antibiogram profile of *Escherichia coli, Salmonella* spp., *Vibrio* spp., *Bacillus* spp., and *Staphylococcus* spp. against five antibiotics. *E. coli* was sensitive to 3 and resistant to 2 antibiotics, *Salmonella* spp. was sensitive to 2 and resistant to 3 antibiotics, *Vibrio* spp. was sensitive to 3 and resistant to 2 antibiotics, *Bacillus* spp. was totally sensitive to 5 antibiotics, and *Staphylococcus* spp. was sensitive to 3 and intermediately sensitive to 1 antibiotic but was resistant to 2 antibiotics.

Summary of antibiogram profile of *E. coli, Salmonella* sp., *Vibrio* sp., *Bacillus* sp., and *Staphylococcus* sp. against antibiotics is presented in Figure-6.

## Discussion

Consumption of ready-to-eat (RTE) food items including betel leaf with food-borne bacteria is an important obstacle in international trade as well as public health issue worldwide. The present research work was undertaken to measure bacterial loads and identification of bacteria in betel leaf sold at local markets in Mymensingh city.

In this study, TVC of bacteria in betel leaf sample sold at different local markets ranged from log 7.403 CFU/ml to log 7.718 CFU/ml which was higher than the permissible limit (10-100 CFU/ml). Higher TVC recorded in this study might be resulted from lack of hygienic measures during cultivation, harvesting, transportation, and selling of betel leaf. A study conducted in restaurant salad vegetables in Chittagong city recorded 5.20-6.87 log CFU/g TVC [11]. In Nigeria, TVC of RTE salad vegetables was ranged from 6.20 to 8.47 log CFU/g [12].

In this study, the highest TVC of betel leaf was found at Shesh more (log 7.718 CFU/ml) and the lowest TVC was recorded at Jobber more (log 7.403 CFU/ml). The variation of TVC in betel leaf sold at local markets is statistically significant (p<0.05). This higher TVC of betel leaf might be due to unsanitary environment, use of polluted water to wash betel leaf, and unclean utensil used to storage betel leaf.

In this study, five different genera of bacteria were isolated from betel leaf. The prevalence of *Salmonella* spp. was the highest (25.52%), and *E. coli* prevalence was the lowest (17.34%). Fakruddin *et al*. (2017) found that 77% betel leaf sample collected from different markets of Dhaka city was found to be contaminated with *Salmonella* spp. [13]. A study conducted in India isolated from RTE betel leaves [14]. Mishra and Mishra [15] isolated *Xanthomonas campestris* pv. *Betticola* bacteria from diseased betel leaves. In a separate study conducted by Sharmin [16], isolated *E. coli, Salmonella* spp., *Vibrio* spp., and *Bacillus* spp. from RTE salad vegetables sold at different local markets in Mymensingh city. *E. coli, Salmonella* spp., *Vibrio* spp., *Bacillus* spp., and *Staphylococcus* spp. are some of food-borne pathogen associated with food-borne infection and intoxication. Foods can be contaminated at many points in the food preparation, storage, and handling process. In this study, betel leaves might be contaminated with bacteria due to the use of dirty water for washing, handling of betel leaves with unclean hands, and use of unclean utensil or cutting board when preparing RTE betel leaves.

In this study, various selective media were used for isolation of *E. coli* from samples. Colony characteristics of *E. coli* observed in EMB were similar to the findings of Sharada *et al*. [17]. Morphologically, *E. coli* was Gram-negative short rod arranged in single or paired and motile. Thomas *et al*. [18] found similar cultural, staining, and motility characteristics of *E. coli*. The identified bacteria were reconfirmed by sugar fermentation and other biochemical tests which were found similar with the findings of Thomas *et al*. [18].

In this study, the colony characteristics of *Vibrio* spp. on TCBS agar plate were similar to the findings of Khan *et al*. [19]. In Gram’s staining, bacteria exhibited curved rod-shaped appearance which was also observed by other researchers [20, 21]. In this study, *Vibrio* spp. fermented dextrose, maltose, mannitol, and sucrose with the production of acid but gave a negative result to VP test [21].

In this study, the colonies of *Salmonella* spp. on XLD agar plate were opaque, translucent with black centers which were similar to the findings of Cheesbrough [22]. In Gram’s staining, *Salmonella* spp. exhibited short rods, Gram-negative, single, or paired in arrangement. Similar findings were also reported by Buxton and Fraser [23]. Sugar fermentation tests profile of *Salmonella* spp. in the present study showed similarities with the findings of Cowan [24]. Morphology and staining characteristics of *Bacillus* spp. recorded in this study are in agreement with the findings of Merchant and Packer [25]. *Bacillus* spp. fermented five basic sugars with the production of acid [26].

Colony characteristics of *Staphylococcus* spp. observed in MS agar were similar to the findings of Chatterjee *et al*. [27] who recorded golden, yellow, and white color colonies. *Staphylococcus* spp. isolates in this study fermented glucose, maltose, lactose, sucrose, and mannitol fermentation with only acid production. These findings are in close agreement with the findings of Chatterjee *et al*. [27].

In this study, antibiotic sensitivity assay showed that all bacterial isolates were resistant to ampicillin. Four isolates such as *E. coli, Salmonella* spp., *Vibrio* spp., *Bacillus* spp., and *Staphylococcus* spp. exhibited multidrug-resistant profile. *Salmonella* spp., in this study, showed resistant against three antibiotics. Singh *et al*. [14] isolated multidrug-resistant *Salmonella* from RTE betel leaves in North Indian cities. In this study, *Salmonella* spp. was found sensitive to ciprofloxacin and resistant to chloramphenicol. *Salmonella* spp. isolated from betel leaves was found sensitive to both ciprofloxacin and chloramphenicol [14]. Indiscriminate use of antibiotic is responsible for the emergence of multidrug-resistant *E. coli* [28].

## Conclusion

Data of this study suggest that betel leaves sold at local markets of Mymensingh city harbor multidrug-resistant bacteria which underscore the need of implementation of hygienic practices during production, harvesting, transportation, storage, selling, and preparation of betel leaves to safeguard public health.

## Authors’ Contributions

MMK and MAI designed the study. MMH and MARS collected and processed the samples for isolation and identification of bacteria. MMH, MARS, and RAR were done PCR and electrophoresis. MMH and MMK interpreted the results and analyzed the data. MMH, MAI, and MMK prepared the manuscript. All authors read and approved the final manuscript.
